# T Follicular Regulatory Cell Suppression of T Follicular Helper Cell Function Is Context-Dependent *in vitro*

**DOI:** 10.3389/fimmu.2020.00637

**Published:** 2020-04-17

**Authors:** Maria Lopez-Ocasio, Maja Buszko, Melissa Blain, Ke Wang, Ethan M. Shevach

**Affiliations:** Laboratory of Immune System Biology, National Institute of Allergy and Infectious Diseases, National Institutes of Health, Bethesda, MD, United States

**Keywords:** T regulatory cell, T follicular helper cell, B cell receptor, germinal center, antibody production

## Abstract

The production of antibody-secreting plasma cells and memory B cells requires the interaction of T follicular helper (Tfh) cells with B cells in the follicle and is modulated by T follicular regulatory (Tfr) cells. We compare the effects of Tfr cells in an *in vitro* model of bystander Tfh function in the absence of BCR engagement and in a model in which mimics cognate T-B interactions in which the BCR is engaged. In the absence of Tfr cells, Tfh cells from primed mice induce naive B cell differentiation into GC B cells and class switch recombination (CSR) in the presence of anti-CD3 alone or anti-CD3/IgM in a contact-dependent manner. Addition of primed Tfr cells efficiently suppressed GC B cell proliferation, differentiation and CSR in the anti-CD3 alone cultures, but only moderately suppressed BCR-stimulated B cells. When stimulated with anti-CD3 alone, IL-4 is critical for the induction of GC B cells and CSR. IL-21 plays a minimal role in GC B cell differentiation, but a greater role in switching. When the BCR is engaged, IL-4 is primarily required for switching and IL-21 only modestly affects switching. CD40L expression was critical for Tfh-mediated B cell proliferation/differentiation in the absence of B cell engagement. When the BCR was engaged, proliferation of CD40 deficient B cells was partially restored, but was susceptible to suppression by Tfr. These studies suggest that *in vitro* Tfr suppressor function is complex and is modulated by BCR signaling and CD40-CD40L interactions.

## Introduction

A crucial aspect of T-cell dependent immunity is the formation of the germinal center (GC) within the B cell follicles of secondary lymphoid organs. It has long been known that these responses are initiated with the migration of T cells, more specifically T follicular helper (Tfh) cells, and B cells (1, 2). Naïve CD4^+^ T cells are localized in the T cell zone of lymphoid organs and TCR engagement with peptide/MHCII on antigen presenting cells (APC), together with the cytokines IL-21 and IL-6, results in the upregulation of CXCR5 allowing these T cells to home toward the follicle (3, 4). Normal development of Tfh cells is required for optimal formation of antibody responses primarily through the active germinal center reaction. Tfh cells express other co-stimulatory molecules such as ICOS and CD40L, that are essential for B cell activation and differentiation, as their absence leads to disrupted GC responses and impaired B cell memory (5, 6). Tfh cells promote the germinal center reaction through IL-4 and IL-21 secretion, helping B cells differentiate into GC B cells, which then undergo class switch recombination (CSR) and somatic hypermutation resulting in terminal differentiation of the GC B cells into plasma cells (5, 6).

A subset of CD4^+^ T cells has been identified within the follicle that expresses Foxp3 and has been termed T follicular regulatory (Tfr) cells. Tfr cells suppress the function of the Tfh cells, reduce the GC reaction, and inhibit antibody responses (7–10). However, the mechanism of action or the cellular target of the Tfr cells remains unclear. Some studies suggest that the major role of Tfr cells is to suppress IL-21 secretion by Tfh cells, as B cell responses could be rescued by the addition of IL-21 to co-cultures of Tfh, primed B cells and Tfr cells (11–14). Other studies have shown that CTLA-4 has a critical role in Tfr-mediated suppression (15, 16). CD40-CD40L interactions also play a critical roles in both the activation of Tfh cells and in the interaction of the activated Tfh cells with B cells and APC. Deficiency of the CD40L results in impairment of T-cell mediated immunity, failure of B cell maturation, isotype switching, and Ab production (17). T_*FR*_ cells have been found to regulate early and not late GC responses to control antigen-specific antibody and B cell memory (18, 25). Signaling thru CD40 has been shown to required for the first wave of BCL6 protein, but it must cease at the next stage to allow for GC B cell progression (19, 26). Therefore evaluating the role of Tfr cells in controlling the early aspects if GC B cells is of importance.

In this report, we have developed a co-culture system using primed Tfh cells and naïve B cells to explore the different suppressive mechanisms used by Tfr cells during GC responses *in vitro*. We compare the effects of Tfr cells in a model of bystander Tfh function in the absence of BCR engagement and in a model in which mimics cognate T-B interactions in which the BCR is engaged. We demonstrate that Tfr cells can suppress Tfh cells *in vitro* primarily by blocking the secretion of IL-4 and to a lesser extent IL-21. In addition to the suppression of cytokine production by Tfh cells, CD40L expression by Tfh is shown to be critical for Tfh-mediated B cell proliferation and B cell differentiation in the absence of B cell engagement. CD40-CD40L interactions were also required for Ig production, but not differentiation, in the presence of B cell engagement. Tfr cells can also directly suppress some aspects of B cell differentiation in a T-cell independent fashion raising the possibility that Tfr cells can directly suppress T-independent pathways of B cell differentiation.

## Materials and Methods

### Mice

C57BL/6 mice were purchased from Charles River. CD40 deficient (^–/–^) mice on the C57BL/6 background were purchased from Jackson laboratories (Bar Harbor, ME). IL-21R^–/–^, IL-4 gfp/gfp and Foxp3-EGFP mice were obtained by the National Institute of Allergy and Infectious Diseases (NIAID) under contract with Taconic Farms (Germantown, NY, United States). All animals were maintained under specific pathogen free conditions and all animal protocols used in this study were approved by the NIAID Animal Care and Use Committee.

### Media, Antibodies, and Reagents

Cell cultures were performed using RPMI 1640 (Lonza) supplemented with 10% heat-inactivated FBS, 100 U/ml penicillin, 100 U/ml streptomycin, 2 mM glutamine and 50 mM 2-ME. The following staining reagents were used for flow cytometry: APC anti-IgG1 (X56) from BD Biosciences (San Jose, CA); BV650 anti-CD138 (281-2), eFluor 710 anti-IgD (11-26c), BV421 anti-CXCR5 (L138D7) from BioLegend (San Diego, CA) from Biolegend. PE anti–PD-1 and APC anti-PD-1 (J43), APC-Cy7 anti-CD4 (RM4-5), PE-Cy7 anti-CD44 (IM7), PE anti-CD25 (PC61), APC anti-CD45 RB (MB4B4), PE anti-CD95 (15A7), anti-CD19 PercP-Cy5.5 (eBio1D3), Alexa Fluor 488 anti-GL7 (GL7), BV421 anti-B220 (RA3-6B2), eFluor anti-IgM (11/41) all purchased from eBiosciences (Thermo Fisher Scientific, Waltham, MA, United States). For magnetic cell separation, we used anti-CD4 beads (LT34, Miltenyi, Bergisch Gladbach, Germany), biotinylated anti-CD43 (S7, BD Pharmingen, San Jose, CA, United States), biotinylated anti-GL7 (GL7, eBiosciences), and biotinylated anti-CD11c (N418, eBiosciences). Intracellular staining was performed with the eBioscience Foxp3 Staining Buffer Set (Thermo Fisher Scientific, Waltham, MA, United States), according to the manufacturer’s protocol.

### Flow Cytometry and Sorting

Cell proliferation was assayed with eBioscience Cell Proliferation Dye eFluor_*TM*_ 450 (Thermo Fisher Scientific, Waltham, MA, United States), according to the manufacturer’s protocols. Cells were allowed to proliferate for 72 h and stained for live cells and cell surface markers. Flow cytometry was performed on a LSR-Fortessa (BD) and analyzed using FlowJo software (BD Biosciences). Cells were sorted on an ARIA III (BD, San Jose, CA, United States).

### Immunization

Mice were immunized in their flanks with 100 μg of myelin oligodendrocyte glycoprotein (MOG_35__–__55_, Protein Chemistry Core, NIAID) emulsified in Complete Freund’s Adjuvant (CFA, Sigma-Aldrich) referred to as MOG/CFA). Draining lymph nodes (dLNs) were harvested 7 d post immunization, cell suspensions were depleted of RBC by lysis buffer and cells were processed for CD4 enrichment (AutoMacs, Miltenyi), followed by surface staining and sorting.

### *In vitro* Tfr Suppression Assay

The *in vitro* suppression assay was performed as previously reported (20, 21) with some modifications. Briefly, Tfh, Tfr and naïve B cells were sorted to 95% purity on an BD FACS Aria III (BD Biosciences, San Jose, CA) based on their expression of cell surface molecules. Naïve B cells (5 × 10^4^), Tfh cells (3 × 10^4^) in the presence or absence of Tfr cells (1.5 × 10^4^) were cultured for 96 h with 2 μg/ml soluble anti-CD3 (2C11; BioXcell) and 5 μg/ml AffiniPure F(ab’)_2_ Fragment Goat Anti-Mouse IgM, μ chain specific (Jackson Immunoresearch; AB_2338469). Control B cells were stimulated with soluble anti-CD40 (3/23, BD Pharmingen). The cultured cells were harvested and analyzed for differentiation by flow cytometry. Proliferation of cells was measured after 72 h by dilution of eFluor 450 cell proliferation dye by flow cytometry. anti-IL-4 (10 μg/ml 11B11) and anti-IL-21 (10 μg/ml, FFA21, eBiosciences) were added to some cultures. For reversal suppression assays, recombinant IL-4 (200 ng/ml) and recombinant IL-21 (2 ug/ml Peprotech) were added to the cultures.

### Transwell Assays

Corning 0.4 μm HTS Transwell-96 Well Permeable Support System (Thermo Fisher) plates were used to assay for cell contact-dependence. Naïve B and Tfh cells were co-cultured in the presence or absence of Tfr cells (see Tfr suppression assay).

### Statistical Analysis

All data are mean ± SEM. Comparison of means between groups was done by two-tailed Student t-test and one-way ANOVA when comparing more than two conditions. Differences were considered statistically significant at *p* < 0.05 (Prism, GraphPad). The average number of divisions that cells underwent in culture was calculated as proliferation index using FlowJo software (BD Biosciences).

## Results

### Tfh Cells Uniquely Promote B Cell Proliferation and Differentiation

**FIGURE 1:**
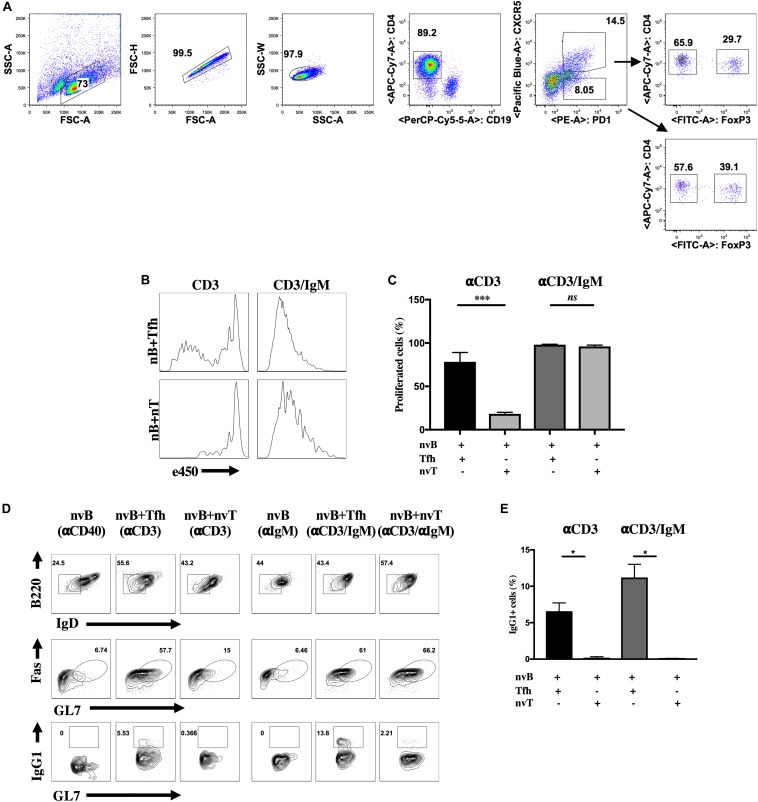
Tfh cells are unique in promoting B cell differentiation and proliferation *in vitro*. **(A)** Sorting strategy for Tfh, Tfr or CXCR5- T reg cells. eGFP-Foxp3 mice were immunized with MOG/CFA and cells were obtained on day 7 post-immunization. Primed Tfh cells and Tfr cells were obtained from dLNs, sorted with CD4 beads, stained and sorted. Tfh cells were identified as CXCR5^+^ PD-1^+^ Foxp3^–^ CD4^+^ CD19^–^, whereas Tfr cells were identified as CXCR5^+^ PD-1^+^ Foxp3^+^ CD4^+^ CD19^–^. CXCR5-Tregs were identified as CXCR5^–^ PD-1^+^ Foxp3^+^ CD4^+^ CD19^–^ (arrow down). Foxp3 was identified by GFP (anti-CD25 and anti-CD44 was added when sorting for IL4-/- Tfh cells). **(B,C)** nvB were labeled with eF450 and cultured with primed Tfh or nvT cells and stimulated with anti-CD3 alone anti-CD3/anti-IgM. B cell proliferation was assayed by dilution of e450. **(D)** nvB cells alone or together with primed Tfh or nvT cells were stimulated with anti-CD3 alone or anti-CD3/anti-IgM. GC B cells were gated as live Fas^+^GL7^+^B220^+^ IgD^*lo*^ B220^+^IgD^–^. **(E)** Switching was measured by staining GC B cells intracellularly with anti-IgG1. Summary of 10 independent experiments measuring IgG1^+^ GC B cells. Statistical results are from 10 independent experiments showing SEM (*n* = 10). *p < 0.05, ***p < 0.0001, unpaired Student *t*-test.

The major goal of this study was to identify the mechanism (s) of suppression utilized by Tfr cells. As an initial approach, we modified an *in vitro* co-culture system to mimic the generation of the *in vivo* GC response (20, 21). To efficiently generate a higher yield of both Tfh and Tfr cells, Foxp3-eGFP mice were immunized with MOG_35__–__55_ peptide emulsified in CFA. Tfh (CD4^+^ CXCR5^+^ PD-1^+^ Foxp3^–^ CD19^–^) and Tfr (CD4^+^ CXCR5^+^ PD-1^+^ Foxp3^+^ CD19^–^) cells were isolated from dLN 7 d post-immunization by cell sorting. Naïve B (nvB) cells (CD19^+^ B220^+^ IgD^*hi*^ GL7^–^ Fas^–^ CD4^–^) and naïve T (nvT) cells (CD4^+^CD25^–^CD45RB^*hi*^gCD44^*lo*^) were purified from the spleens of unimmunized WT mice by cell sorting. A sorting strategy for the cell populations used in all experiments is shown in Figure 1A and control gating strategy is shown in Supplementary Figure 4. To characterize the role of Tfh cells in promoting B cell proliferation and differentiation *in vitro*, e450-labeled naïve B cells were co-cultured at a ratio of 2:1 with purified Tfh or nvT cells in the presence of anti-CD3 or anti-CD3/anti-IgM for 3 d. nvT cells were unable to induce nvB cell proliferation when cultures were stimulated with anti-CD3 alone. In contrast, a modest degree of nvB cell proliferation was observed in the presence of Tfh cells (Figures 1B,C). Stimulation of nvB cells with anti-IgM alone induced only a minimal proliferative response (data not shown), but anti-CD3/anti-IgM induced vigorous proliferation in the presence of either Tfh or nvT cells (Figures 1B,C). These data suggest that Tfh cells play a unique role in promoting B cell proliferation in bystander fashion in the absence of B cell receptor (BCR) engagement by provided cytokine-mediated help and co-stimulatory signals.

We next compared the ability of nvT cells and Tfh cells to promote nvB cell differentiation as defined by downmodulation of IgD expression together with acquisition of GC B cell surface markers (Fas and GL7), and isotype switching. nvB cells stimulated with anti-IgM downregulated IgD expression, but failed to differentiate to GC B cells (Figure 1D, column 4). Similar results were observed when B cells were co-cultured with nvT cells in the presence of anti-CD3 only (Figures 1D, column 3, E). In contrast, when nvB cells were cultured with Tfh in the presence of anti-CD3 or anti-CD3/anti-IgM, they differentiated into Fas^+^GL7^+^ B cells, define as follicular GC B cells and were also capable of switching into IgG1^+^ B cells (Figures 1D, columns 2 and 5, E). However, stimulation of naïve B cells with anti-CD3/anti-IgM in the presence of nvT cells while resulting in GC B cells differentiation, as based on Fas and GL7 expression, failed to induce isotype switching (Figures 1D, column 6, E). Taken together, these observations suggest that this *in vitro* model is valid for analyzing Tfh cell function in inducing both B cell proliferation, differentiation, and isotype switching.

### Tfh Driven B Cell Differentiation Is Cell Contact-Dependent

**FIGURE 2:**
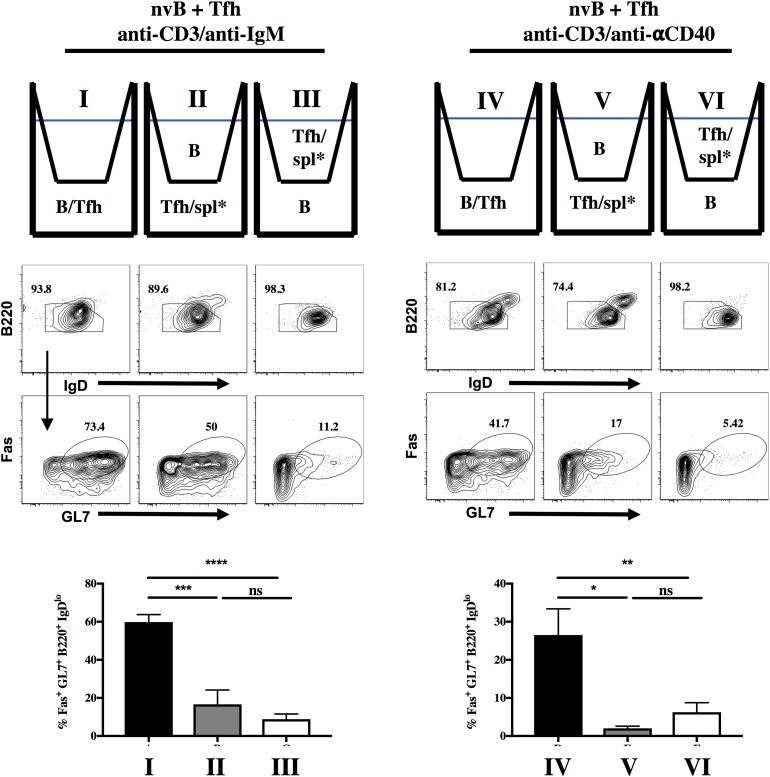
B cell differentiation is dependent on contact with Tfh cells. nvB cells were sorted from B6 mice and co-cultured with Tfh cells, and stimulated with (I–III) anti-CD3/anti-IgM or (IV–VI) anti-CD3/anti-CD40. nvB cells and Tfh cells were gated as live B220^+^CD4^–^ and Tfh cells gated as live CD4^+^ GFP^–^, respectively. Statistical results are from 8 independent experiments showing SEM (*n* = 8). *p < 0.05, **p < 0.005, ***p < 0.0001, ****p < 0.00001, ns: not significant; unpaired Student t test. Spl* = irradiated splenocytes.

We first determined whether Tfh driven B cell differentiation was cell contact-dependent. nvB cells and Tfh cells were cultured together in the lower chamber of a transwell or separated by the transwell membrane and stimulated with anti-CD3/anti-IgM. When the Tfh were separated from the nvB cells, the cultures were supplemented with irradiated splenocytes (spl^∗^) to allow Fc-mediated cross linking of the soluble anti-CD3. Separation of the Tfh cells from the B cells did not inhibit downregulation of IgD expression, but markedly inhibited the induction of GC B cell differentiation (Figure 2, I–III). Similar results were observed when the co-cultures of Tfh and nvB cells were stimulated with anti-CD3/anti-CD40 (Figure 2, IV–VI). These observations showed that B cell differentiation during the *in vitro* generation of antibody responses is dependent on Tfh-B cell contact.

### Tfr Cells Directly Suppress Tfh Cell Proliferation *in vitro*

**FIGURE 3:**
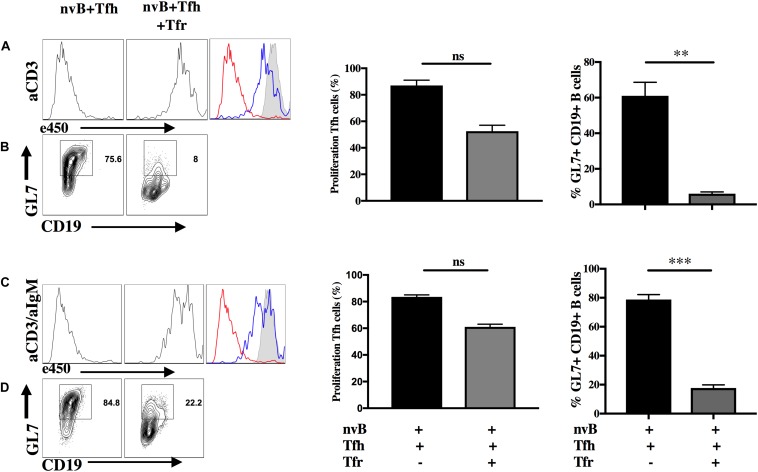
Tfr cells directly suppress Tfh cell proliferation *in vitro.* nvB cells and primed Tfh cells were cultured in the presence (blue) or absence (red) of primed Tfr and stimulated with **(A,B)** anti-CD3 alone or **(C,D)** anti-CD3/anti-IgM for 3 d. Tfh cells were labeled with eF450 cell tracker according to manufacturer instructions prior to culture and assay for suppression of proliferation. CD19^+^ B cells were analyzed for GL7 expression, statistics were calculated and shown on the right. Statistical results are from five independent experiments showing SEM (*n* = 5). **p < 0.005, ***p < 0.0001, unpaired Student *t*-test.

To begin to determine the target cell(s) of Tfr-mediated suppression of Tfh induced B cell differentiation, we labeled Tfh cells with a cell tracker (e450) and cultured them with nvB cells in the presence or absence of Tfr cells. Tfh cells proliferated vigorously when stimulated with anti-CD3 in the presence of nvB cells and their proliferation was almost completely abrogated in the presence of Tfr cells (Figure 3A). When we examined the B cells in these same cultures, B cell differentiation as measured by GL7 expression was also markedly inhibited (Figure 3B, graph). Similarly, Tfh cell proliferation was also almost completely inhibited when the cultures were stimulated with anti-CD3/anti-IgM (Figure 3C). However, B cell differentiation as measured by GL7 expression, was only partially inhibited when on the anti-CD3/anti-IgM compared to anti-CD3 conditions alone (Figure 3D, graph). Taken together, these studies demonstrate that Tfh proliferation is required to induce B cell differentiation and that Tfh cell proliferation and B cell differentiation are completely inhibited by Tfr cells. However, in the presence of engagement of the BCR, GC differentiation is partially resistant to Tfr mediated suppression.

### Tfr Cells Suppress Tfh-Induced B Cell Proliferation

**FIGURE 4:**
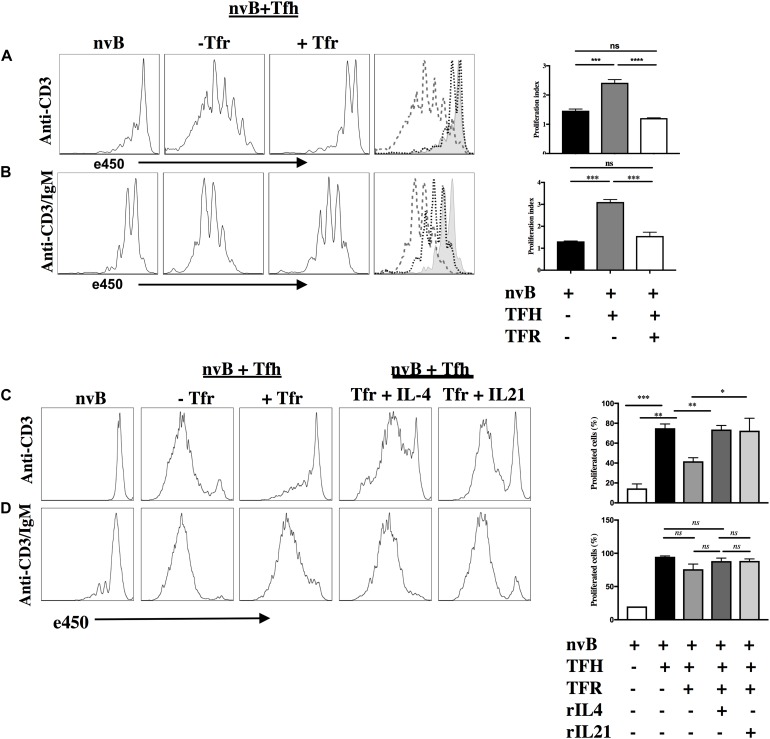
Tfr cells suppress B cell proliferation in a T-cell dependent manner. **(A)** nvB were labeled with eF450 and cultured with primed Tfh cells (dashed), anti-CD3, in the presence or absence of primed Tfrs (dotted). **(B)** e450-labeled nvB cells were cultured with primed Tfh cells, stimulated with anti-CD3/anti-IgM in the presence of absence of Tfr cells. **(C)** As in panel **(A)**, except IL-4 or IL-21 were added to cultures with Tfr cells. **(D)** As in panel **(B)** except cultures with Tfr cells were supplemented with IL-4 or IL-21. Control B cells were stimulated with anti-IgM (5 μg/ml) alone. Statistical results are from 10 independent experiments showing SEM (*n* = 10). *p < 0.05, **p < 0.005, ***p < 0.0001, ****p < 0.00001, ns: not significant; unpaired Student *t*-test.

We next evaluated the capacity of Tfr cells to suppress B cell proliferation induced by Tfh cells. When nvB cell were cultured with Tfh cells and stimulated with anti-CD3 alone, B cell proliferation was almost completely abrogated by Tfr cells (Figure 4A). In contrast, when nvB cells were stimulated with Tfh cells and anti-CD3/anti-IgM, significant inhibition of B cell proliferation was observed, but the magnitude of inhibition was less than in cultures stimulated with anti-CD3 alone (Figure 4B). Tfr cells were more potent inhibitors of B cell proliferation than non-CXCR5 Treg (Supplementary Figure 1). These data are consistent with the possibility that Tfr cells primarily act by inhibiting B cell stimulatory cytokine production by Tfh cells or by inhibiting the induction of the CD40L expression on Tfh cells which is required for CD40-CD40L interactions and induction of B cell proliferation. In contrast, when the BCR is engaged by anti-IgM, a component of the B cell proliferative response as well as GC B cell differentiation (Figure 3D) is resistant to Tfr-mediated inhibition.

To determine the contribution of Tfh-derived cytokines to B cell proliferation, we attempted to reverse Tfr-mediated suppression of B cell proliferation by adding cytokines back to the co-cultures. When Tfh driven B cell proliferation in the presence of anti-CD3 was inhibited in the presence of Tfr, the addition of either IL-4 or IL-21 partially restored the B cell response (Figure 4C). In contrast, when nvB cells were stimulated with Tfh cells and anti-CD3/anti-IgM, modest suppression of B cell proliferation was again observed, but the addition of IL-4 or IL-21 had little to no effect on the B cell proliferative response (Figure 4D). Taken together, these data suggest that in the absence of BCR ligation, Tfh driven B cell proliferation is substantially Tfh-derived cytokine dependent and readily suppressed by Tfr cells. In contrast, in the presence of BCR ligation, a substantial fraction of the B cell response is not cytokine driven and is resistance to Tfr-mediated suppression.

### Tfr Cells Suppress B Cell Activation and Differentiation by Inhibiting IL-4 and IL-21 Production by Tfh Cells

**FIGURE 5:**
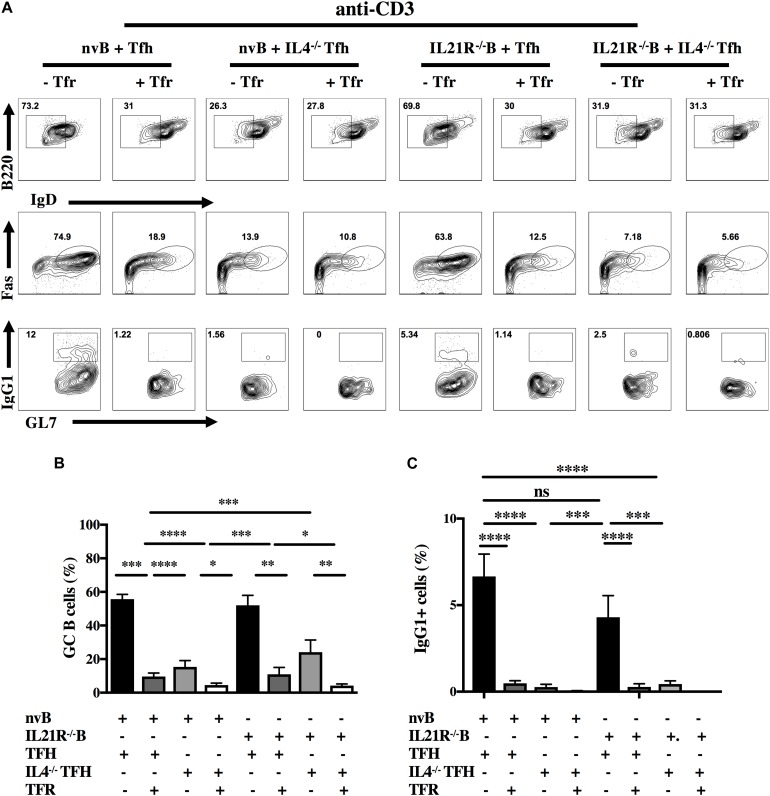
Role of IL-4, IL-21, and Tfr on GC B cell induction and class switching. **(A)** nvB cells from unimmunized B6 and IL-21R^–/–^ mice were co-cultured with primed Tfh cells from either Foxp3-eGFP or IL4^–/–^ mice and stimulated with anti-CD3 in the presence or absence of primed Tfr cells. **(B)** GC B cells were gated as live Fas^+^GL7^+^B220^+^IgD^*lo*^ CD4^–^. **(C)** IgG1^+^ cells were gated as live B220^+^ IgD^*lo*^CD4^–^. Statistical results are from 10 independent experiments showing SEM (*n* = 10). *p < 0.05, **p < 0.005, ***p < 0.0001, ****p < 0.00001, unpaired Student *t*-test and ANOVA.

Both IL-4 and IL-21 have been implicated in the regulation of the GC reaction (11, 28, 29). To evaluate the contribution of these cytokines in our model, we isolated Tfh cells from animals deficient in IL-4 (IL-4^–/–^) and nvB cells from animals deficient in the IL-21 receptor (IL-21R^–/–)^ (30). A strategy for sorting IL4^–/–^ Tfh cells is shown (Supplementary Figure 3). When nvB cells were cultured with IL4^–/–^ Tfh under bystander conditions in the presence of anti-CD3 alone, B cells downmodulated IgD, but failed to differentiate into Fas^+^GL7^+^ GC B cells and failed to switch into IgG1^+^ B cells (Figures 5A–C, column 1–4). While Tfr cells markedly suppressed GC B cell differentiation and IgG1 switching in the presence of WT Tfh cells, they did not produce any additional inhibition when IL-4^–/–^ Tfh cells were present.

When we cultured nvB cells from IL-21R^–/–^ mice with WT Tfh cells in the presence of anti-CD3 alone, IL-21R^–/–^ naïve B cells differentiated into GC B cells as efficiently as WT B cells although their capacity to switch to IgG1 was moderately impaired (Figures 5A–C, column 5–8). Tfr cells markedly inhibited GC B cells differentiation and switching when added to cultures of WT Tfh cells and IL-21R^–/–^ B cells. The results of culture of IL4^–/–^ Tfh and IL-21R^–/–^ B cells resembled the results observed with IL-4^–/–^ Tfh cells (Figures 5A–C, column 7–8) indicating that the effects of the Tfr in the presence of IL-21R^–/–^ B cells were mediated by inhibition of IL-4 production by the Tfh.

**FIGURE 6:**
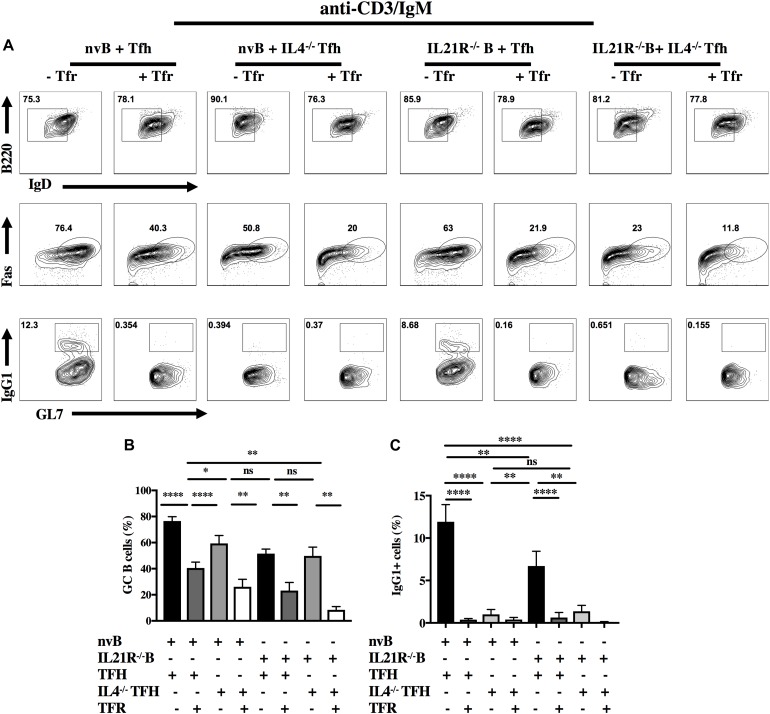
Role of IL-4, IL-21, and Tfr on GC B cell induction and CSR in the presence of BCR engagement. **(A)** nvB from unimmunized B6 and IL-21R^–/–^ mice were co-cultured with primed Tfh cells from either Foxp3-eGFP or IL4^–/–^ mice and stimulated with anti-CD3/anti-IgM in the presence or absence of primed Tfr cells. **(B)** GC B cells were gated as live Fas^+^GL7^+^B220^+^IgD^*lo*^ CD4^–^. **(C)** IgG1^+^ cells were gated as live B220^+^ IgD^*lo*^CD4^–^. Statistical results are from 10 independent experiments showing SEM (*n* = 10). *p < 0.05, **p < 0.005, ****p < 0.00001, unpaired Student *t*-test.

When nvB cells were cultured with IL4^–/–^ Tfh cells and stimulated with anti-CD3/anti-IgM, they normally downmodulated IgD expression, but had a 30% reduction in GC B cell differentiation (83–52%), and failed to switch into IgG1^+^ cells (Figures 6A–C, column 1–4). The addition of Tfr cells further decreased GC differentiation. When nvIL-21R^–/–^ B cells were cultured with WT Tfh cells and stimulated with anti-CD3/anti-IgM, the IL-21R^–/–^ B cells, downmodulated IgD expression normally, but exhibited only a slight reduction in the frequency of GC B differentiation (83–68%), but a somewhat greater reduction in the frequency of IgG1^+^ B cells (11.9–7%) when compared to WT B cells cultured with WT Tfh cells (Figures 6A–C, column 1 vs. 5). Stimulation of IL4^–/–^ Tfh and IL-21R^–/–^ B cells with anti-CD3/anti-IgM resulted in a minimal induction of GC B cell differentiation, and no IgG1 isotype switching (Figures 6A–C, column 7). Addition of Tfr cells to these cultures further suppressed the induction of GC B cells (Figures 6A–C, column 8).

### Role of CD40-CD40L Interactions in GC B Cell Differentiation

The CD40L (CD154) is rapidly induced after TCR stimulation and CD40-CD40L interactions play an important role in antibody production (17). In addition to the contribution of Tfh-derived cytokines to GC B cell differentiation, we used our co-culture system to determine the contribution of CD40-CD40L interactions to B cell proliferation/differentiation *in vitro*. First, we measured the ability of CD40^–/–^ B cells to proliferate in the presence of WT Tfh cells. CD40^–/–^ B cells failed to proliferate when stimulated with anti-CD3 alone (Supplementary Figure 2). Furthermore, when CD40^–/–^ B cells were stimulated in the presence of anti-CD3/anti-IgM, their proliferation was significantly less than that seen with WT B cells. Most importantly, the proliferative response of CD40^–/–^ B cells under the latter conditions was completely inhibited by Tfr cells, while the response of WT B cells was only slightly inhibited by Tfr cells (Supplementary Figure 2 and Figure 4D).

**FIGURE 7:**
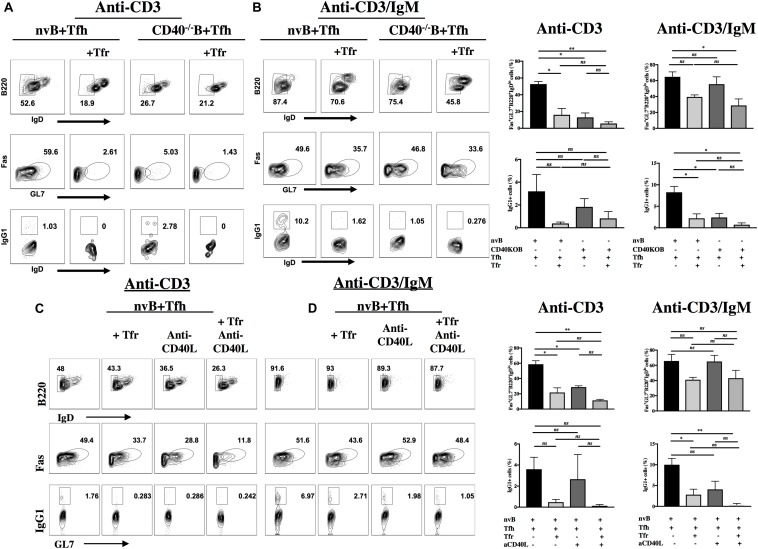
Role of CD40L-CD40 interactions in induction of GC B cells and class switching. nvB cells from unimmunized WT or CD40^–/–^ mice were co-cultured with primed Tfh cells in the presence or absence of primed Tfr cells. Cultures were stimulated with anti-CD3 **(A,C)** or anti-CD3/IgM **(B,D)**. Anti-CD40L was added to cultures in panels C and D. Differentiated B cells were gated as Fas^+^GL7^+^ B220^+^ IgD^*lo*^ CD4^–^ and switched B cells were gated as IgG1^+^GL7 + IgD^*lo*^B220^+^CD4^–^. **(D)** Statistical results are from five independent experiments showing SEM (*n* = 5). *p < 0.05, **p < 0.005, unpaired Student *t*-test.

Next, we evaluated B cell differentiation in the absence of the CD40 signaling. While nv WT B cells differentiated to GC B cells and isotype switched when cultured with WT Tfh cells and anti-CD3 alone, the differentiation of CD40^–/–^ B cells was markedly impaired (Figure 7A) (59.6% vs. 5%) and no difference in switching was seen (Figure 7A) (1.03% vs. 2.7%). Addition of Tfr cells to these cultures further suppressed the induction of GC B cells (2.6–1.4%). In contrast, CD40^–/–^ B cells differentiated normally when stimulated with anti-CD3/anti-IgM and this response (IgG1) was suppressed to a greater extent by Tfr cells than that of WT B cells (Figure 7B), mimicking the results of the B cell proliferation assay. Very similar results were observed when we stimulated WT B cells under identical conditions, but in the presence of anti-CD40L (Figure 7C). The only difference noted was that the GC B cell response induced by anti-CD3/anti-IgM was resistant to inhibition by Tfr cells (51.6–43.6% compared to 52.9%). Nevertheless, comparable suppression of isotype switching by the combination of Tfr and anti-CD40L was seen under the two experimental conditions (0.28–0.2% compared to 2.7–1%) (Figure 7D).

### Tfr Cells Can Directly Target B Cell Differentiation

**FIGURE 8:**
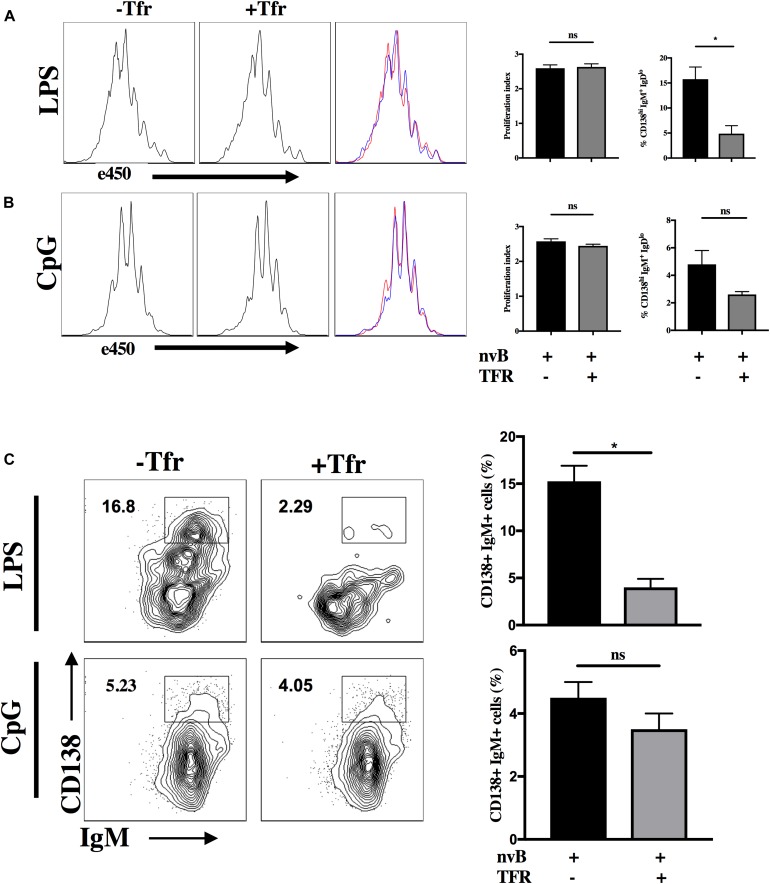
Tfr cells suppress LPS-induced B cell differentiation. **(A)** nvB were labeled with eF450 and cultured for 3 days alone or with Tfr cells in the presence of LPS and anti-CD3 or with CpG and anti-CD3 **(B)**. The average number of divisions that cells underwent in culture was calculated as proliferation index using FlowJo. **(C)** Plasmablasts were gated as CD138^+^ B220^+^ IgM^+^ IgD^*lo*^ cells. Statistical results are from 10 independent experiments showing SEM (*n* = 10). *p < 0.05, unpaired Student *t*-test.

Since our studies (Figure 2) demonstrated that Tfh-B cell interactions were cell contact-dependent, we could not directly determine whether Tfr targeted Tfh or B cells. As one study (22) has claimed that Treg cells can directly inhibit B cell proliferation induced by LPS, we evaluated whether Tfr cells are capable of directly inhibiting T cell independent B cell proliferation. nvB cells were cultured in the presence or absence of Tfr cells and stimulated with LPS or CpG. Both LPS and CpG driven B cell proliferation were completely resistant to inhibition by Tfr cells (Figures 8A,B). We also examined whether Tfr cells would inhibit B cell differentiation when stimulated with LPS. Surprisingly, Tfr cells potently suppressed B cell differentiation as measured by the induction of IgD^*lo*^IgM^+^CD138^*hi*^ IgM-producing plasma cells (18–6%) (Figure 8C). A similar trend of inhibition of plasma cell differentiation was observed when the B cells were stimulated with CpG, but the results were not statistically significant (Figure 8C). It thus appears that Tfr cells can directly act on B cells to partially inhibit differentiation, but not proliferation, but the inhibitory effects may depend on the nature of the stimulus (LPS vs. CpG).

## Discussion

B cell responses in the follicle occur upon interaction of Tfh cells with B cells. Tfh cells provide co-stimulation as well as stimulatory cytokines to B cells leading to affinity maturation, CSR and antibody production which are all necessary for B cell responses. Tfr cells are a subset of Treg cells that upregulate the expression of the chemokine receptor CXCR5 in order to gain access to the B cell follicle where they suppress Tfh cell-mediated B cell responses (25). The mechanisms whereby Tfr cells suppress B cell responses have not been fully characterized and both cell-contact dependent (14, 23) and cytokine mediated mechanisms (14) have been proposed. The goal of this study was to further define the various mechanisms Tfr cells use to suppress B cell responses either indirectly by suppressing Tfh cells or by directly acting on B cells.

We have modified the Tfh-Tfr-B cell co-culture system developed by other groups (20, 21) and used primed Tfh and Tfr cells, but have only used nvB cells as the responder population. In addition, we have compared the effects of Tfr cells on Tfh-mediated B cell responses in the presence and absence of engagement of the BCR. We believe this assay system uniquely measures Tfh function as primed Tfh cells, but not nvT cells, were capable of inducing B cell proliferation. Furthermore, while both Tfh cells and nvT cells could induce the expression of the germinal center B cell markers, FAS and GL7, only Tfh cells were capable of inducing CSR. Plasma cell differentiation in this co-culture system was absolutely dependent on Tfh-B cell contact as B cell differentiation was not observed when the Tfh cells were separated from B cells by a transwell membrane.

We first examined the effects of Tfr cells on Tfh proliferation and observed that Tfr cells could completely inhibit Tfh proliferation induced by anti-CD3 as well as B cell differentiation as measured by GL7 expression. In contrast, when we simultaneously engaged the BCR, T cell proliferation was still completely inhibited, but GL7 expression by B cells was only partially inhibited. Similar results were observed when we examined B cell proliferation in the co-cultures. Tfr cells completely inhibited B cell proliferation in the absence of BCR engagement, but only partially inhibited B cell proliferation in the presence of anti-IgM stimulation. Under bystander conditions with anti-CD3 alone, CSR was always completely inhibited as measured by detection of IgG1^+^ cells (Figure 5). Although GC differentiation was only inhibited by 50% when the BCR was engaged, CSR was still completely blocked (Figure 6). This result is consistent with a model in which B cell activation is dependent on both Tfh derived cytokine production which is readily inhibited by Tfr cells, but that a certain component of the response is resistant to Tfr-mediated inhibition when the BCR is engaged.

Since Tfh derived IL-4 and IL-21 have been implicated (11, 12, 29) as drivers of B cell responses, we tested their contribution to B cell differentiation in our culture system by using Tfh cells from IL-4^–/–^ mice or naïve B cells from IL-21R^–/–^ mice (30). In the absence of BCR stimulation co-culture of IL-4^–/–^ Tfh cells with naïve WT B cells lead to defective GC B cell differentiation and impaired CSR. This result is very similar to what we observed when we cultured WT Tfh cells and B cells in the presence of Tfr cell suggesting that the major effect of Tfr cells in our model is suppression of IL-4 production. On the other hand, co-culture of WT Tfh cells with IL-21R^–/–^ B cells resulted in normal GC B cells differentiation, but a reduced frequency of IgG1^+^ cells. The addition of Tfr cells resulted in complete suppression of both GC B differentiation and CSR likely due to suppression of IL-4 production. A similar result was observed when we co-cultured IL-4^–/–^ Tfh and IL-21R^–/–^ B cells as no further suppression was seen in the presence of Tfr cells. When IL-4^–/–^ Tfh cells were cultured with WT B cells in the presence of anti-IgM, a 40% reduction of B cell differentiation was still observed, but no CSR was seen.

Taken together, these data suggest that IL-4 production by primed Tfh cells stimulated with anti-CD3 alone is critical for the induction of GC B cells and class switching; IL-21 plays only a minimal role in GC B cell differentiation, but a somewhat greater role in switching. Tfr cell appear to block both GC induction and switching by primarily inhibiting IL-4 production by Tfh cells. When the BCR is engaged, IL-4 is primarily required for switching and IL-21 only modestly affects switching. When both IL-4 production and IL-21 responsiveness are blocked, both GC B cell induction and class switching are markedly impaired. Again, the primary effect of the Tfr cells is to inhibit IL-4 production although some Tfr mediated inhibition of the residual GC induction is seen in the absence of both IL-4 and IL-21. These results differ from those of Sage et al. (23) who demonstrated a much more prominent role for IL-21 than IL-4 in Tfh mediated B cell differentiation. One important difference between our studies and those of Sage et al. (14, 20) is that these investigators used total CD19^+^ B cells from immunized mice, while we used naïve B cells from unimmunized animals. The disadvantage of using total CD19^+^ B cells is that this population is heterogenous and contains activated B cells in different stages of differentiation including GC B cells. This difference may account for the much greater contribution of IL-21 in their model.

We have also used our co-culture system to evaluate the role of CD40-CD40L interactions for the promotion B cell proliferation as well as differentiation and CSR. CD40^–/–^ B cells failed to proliferate to TCR derived cytokines when stimulated with Tfh cells and anti-CD3. Furthermore, when stimulated with Tfh cells under anti-CD3/anti-IgM conditions, CD40^–/–^ B cells responded less well than WT B cells. However, the response of CD40^–/–^ B cells was completely inhibited by Tfr, while the response of WT cells was partially resistant to Tfr suppression. As expected, CD40^–/–^ B cells also exhibited a substantial deficit in upregulation of Fas and GL7 when stimulated with Tfh and anti-CD3 and failed to switch, but did differentiate as well as WT B cells when stimulated with anti-CD3/anti-IgM, but failed to switch. As in the proliferation assay, the induction of Fas and GL7 were inhibited to a greater extent with CD40^–/–^ B cells than WT B cells. Taken together, it appears that in the absence of BCR signaling, CD40 signaling is required as a co-stimulatory signal for Tfh-derived cytokines that mediate B cell proliferation. In the presence of BCR signaling, CD40-CD40L interactions play a modest role in co-stimulation of B cells proliferation, but play no role in B cell differentiation. The markedly enhanced susceptibility of the response of CD40^–/–^ B cells to Tfr inhibition suggests that CD40-CD40L interactions induce resistance to Tfr-mediated suppression and that Tfr cells most likely fail to suppress induction of the CD40L on primed Tfh cells.

Previous work by others have shown that Tregs can inhibit B cell activation *in vitro* and *in vivo* in SLE settings and our group have shown in the past that Tregs can significantly suppress B cells (14, 23, 24). Recently, work done by others have shown that Tfr cells can control responses by restraining early B cell responses (25) and the absence or deficiency of these cells can lead to autoimmunity (27). The question of whether Tfr cells can directly target B cells is difficult to address using this co-culture system as the assay requires the presence of both Tfh and Tfr. As an alternative approach, we evaluated whether Tfr cells could directly suppress B cell proliferation and differentiation in the absence of Tfh cells when stimulated with the T independent mitogens, LPS or CpG. Tfr cells had no effect on proliferation induced by LPS or CpG, but LPS-stimulated B cell cultures had reduced frequencies of CD138^+^IgM^+^IgD^*lo*^ plasma cells. A similar trend for a reduction of plasma cells was seen in CpG-stimulated B in the presence of Tfr cells. These results differ markedly from those of Xu et al. (22) who demonstrated that thymic-derived Treg (tTreg) and TGF-β–induced Treg (iTreg) markedly directly suppressed B cell activation and proliferation stimulated by LPS. tTreg appeared to inhibit B cells by a cytotoxic mechanism, while the mechanism of action of iTreg was TGF-β-dependent. It is difficult to compare these results with our studies as Xu et al. (22) used total Treg cells rather than Tfr cells and expanded the cells *in vitro* for several days while we used freshly explanted cells. Curiously, Xu et al. (22) also failed to activated their Treg populations during the co-culture with B cells and did not evaluate the purity of their expanded populations. Other studies (14) have claimed that Tfr cells can directly inhibit B−cell activation, as measured by the attenuation of the levels of GL7 expression or can directly inhibit fully differentiated B cells that have undergone CSR to IgG1 *in vivo*, suggesting direct suppression of class switched effector B cells. While our studies demonstrated Tfr mediated suppression of differentiation, it remains unclear what mechanism Tfr cells might utilize that would result in normal proliferation, but a failure of differentiation. One intriguing possibility is that Tfr cells inhibit cytokine production by LPS activated B cells and cytokines augment the induction of plasma cell formation in this system.

In summary, all of the studies described in this report were performed *in vitro* and it is therefore difficult to fully translate our results to the *in vivo* situation. Furthermore, the activity of Tfr cells is clearly context dependent. While B cells under bystander conditions can proliferate and fully differentiate in the absence of engagement of their BCR and Tfr profoundly suppress this process, the magnitude of Tfr suppression is greatly influenced and reduced when the BCR is engaged. An important pathway mediating this resistance in the presence of BCR signaling is the CD40-CD40L interaction which renders the B cell resistant to Tfr-mediated suppression. Further studies addressing these issues as well as the capacity of Tfr to directly suppress B cells in an *in vivo* model are indicated.

## Data Availability Statement

The raw data supporting the conclusions of this article will be made available upon reasonable request to the corresponding author.

## Ethics Statement

The animal study was reviewed and approved by the Animal Care and Use Committee of the National Institute of Allergy and Infectious Diseases.

## Author Contributions

ML-O made substantial contribution to the conception and design of the study, drafted the manuscript, performed experimental procedures, made substantial contributions to the analysis and interpretation of the data, and reviewed the manuscript critically for important intellectual content. MBi and MBu performed the critical experiments and made substantial contribution to analysis of the data. KW performed all cell sorter experiments and made substantial contributions to the analysis of the data. ES made substantial contribution to the conception and design of the study, drafted the manuscript, made substantial contributions to the analysis and interpretation of the data, reviewed the manuscript critically for important intellectual content, and gave final approval for the version to be published.

## Conflict of Interest

The authors declare that the research was conducted in the absence of any commercial or financial relationships that could be construed as a potential conflict of interest.
