# Supplementary figures and images for: T Follicular Regulatory Cell Suppression of T Follicular Helper Cell Function Is Context-Dependent in vitro
Source: Front Immunol. 2020 Apr 17;11:637. doi: 10.3389/fimmu.2020.00637 (PMC7181357; doi:10.3389/fimmu.2020.00637)

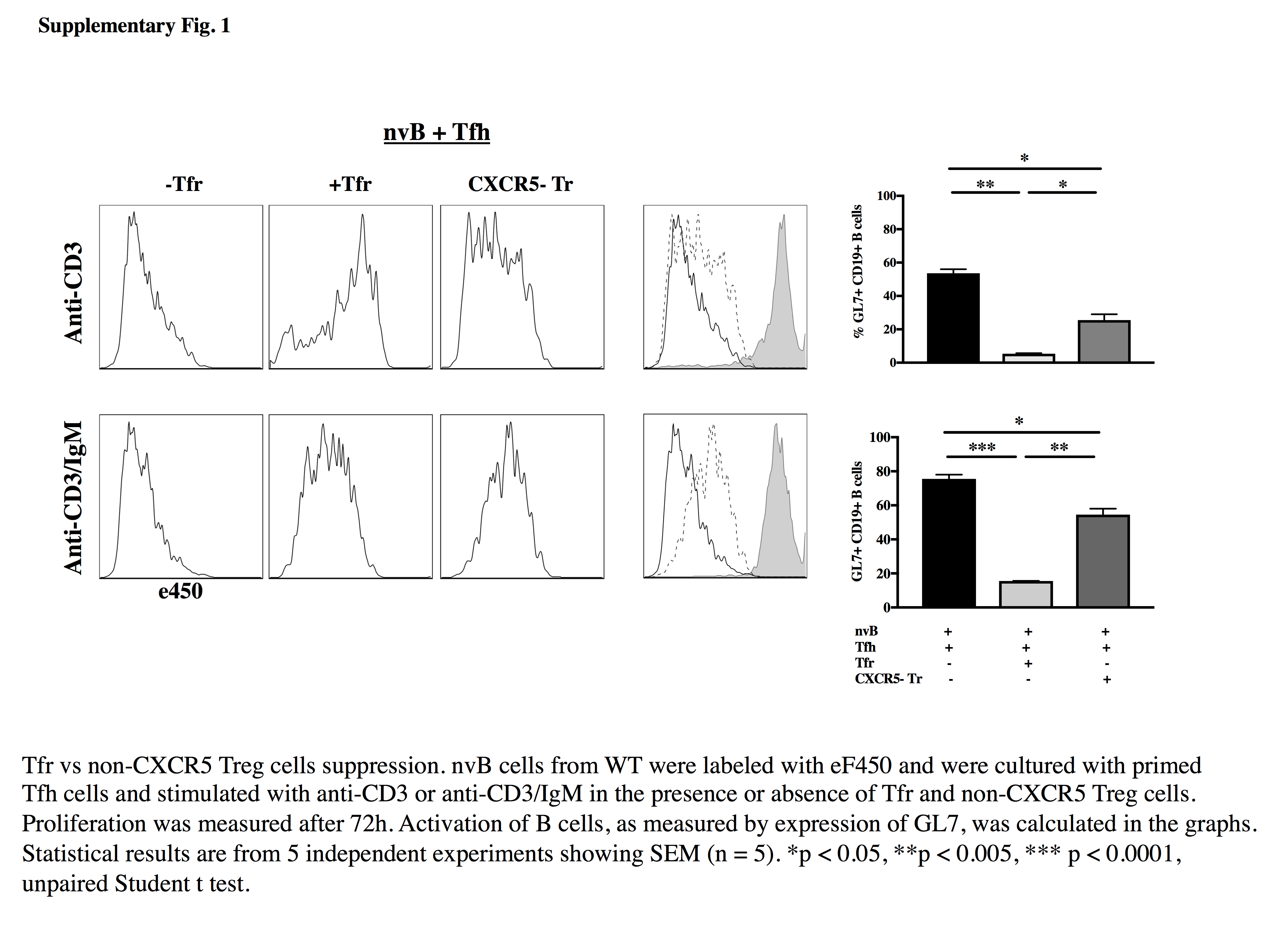

Supplement: Supplementary file 1 [file Image_1.TIFF]

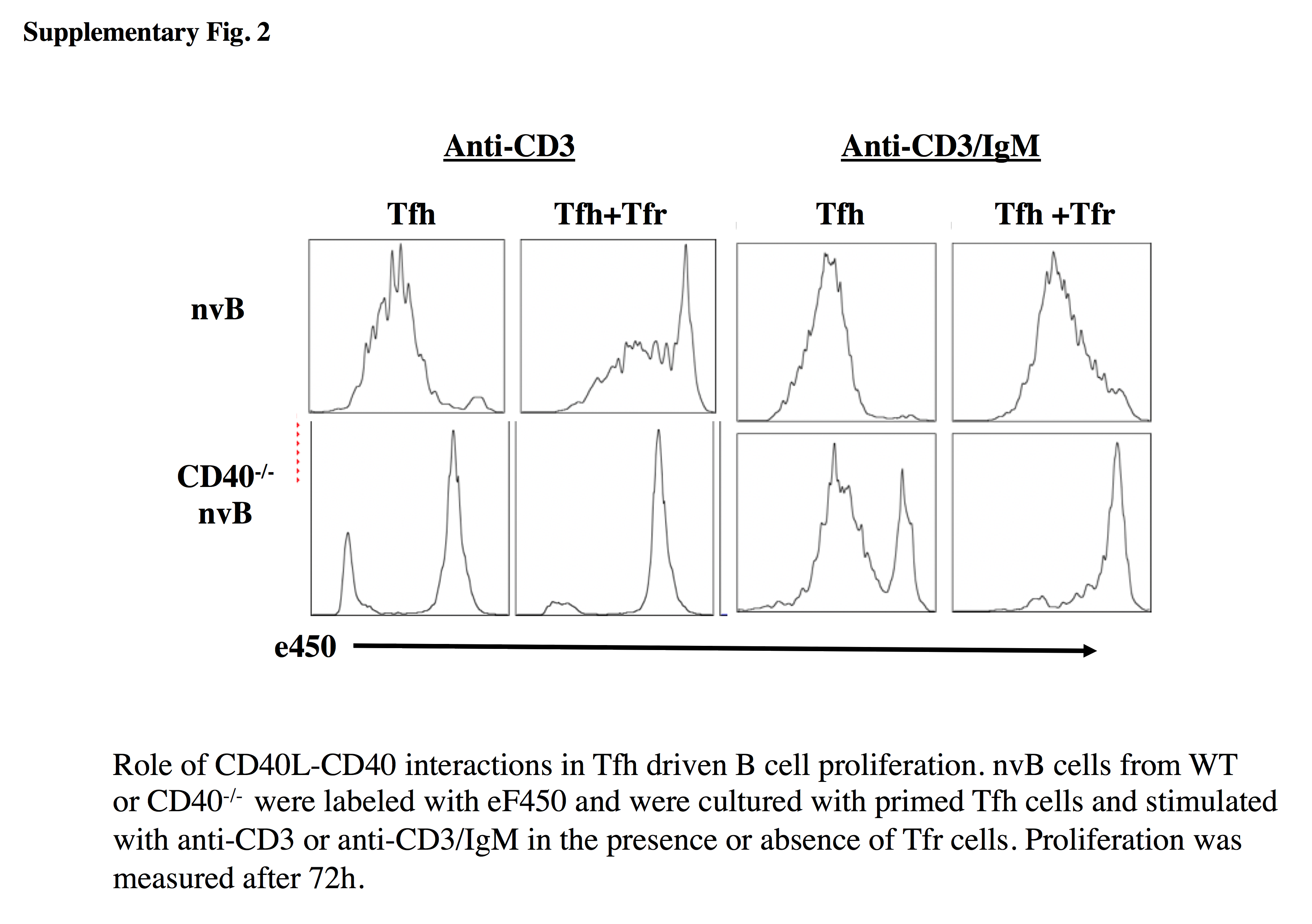

Supplement: Supplementary file 2 [file Image_2.TIFF]

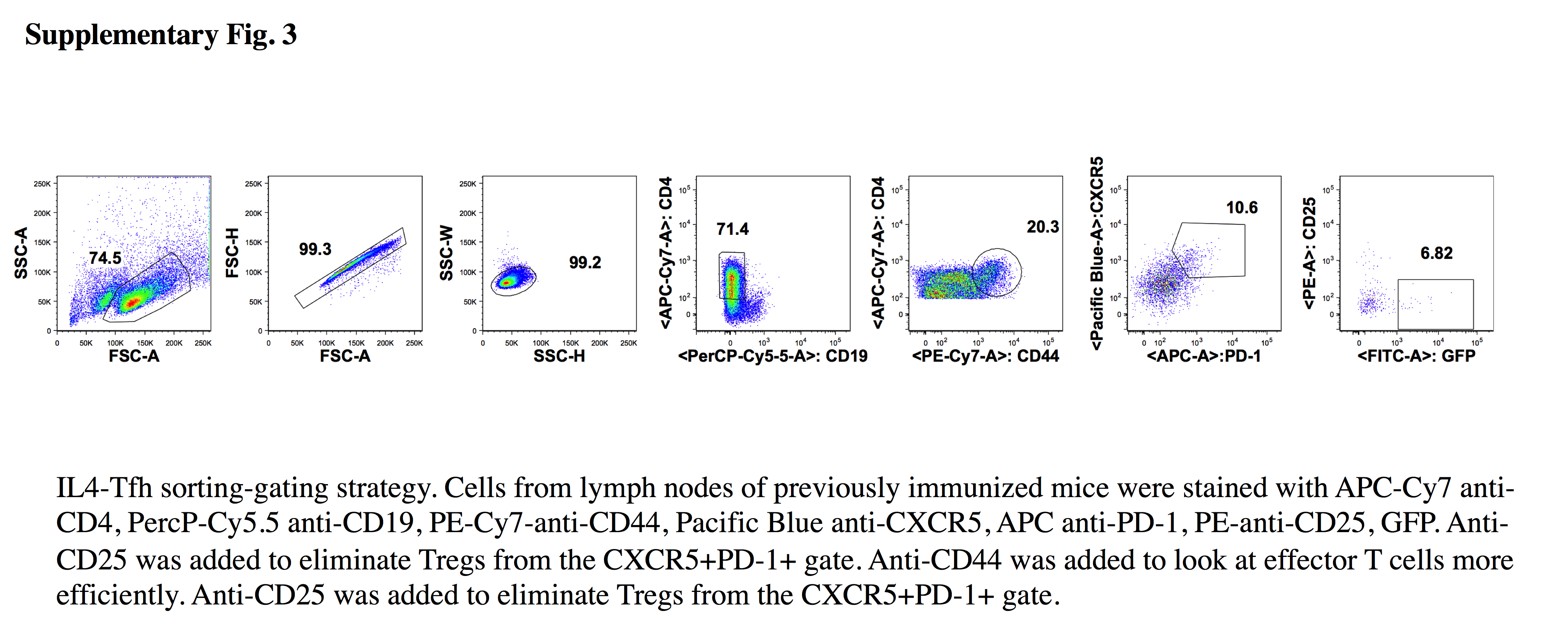

Supplement: Supplementary file 3 [file Image_3.TIFF]

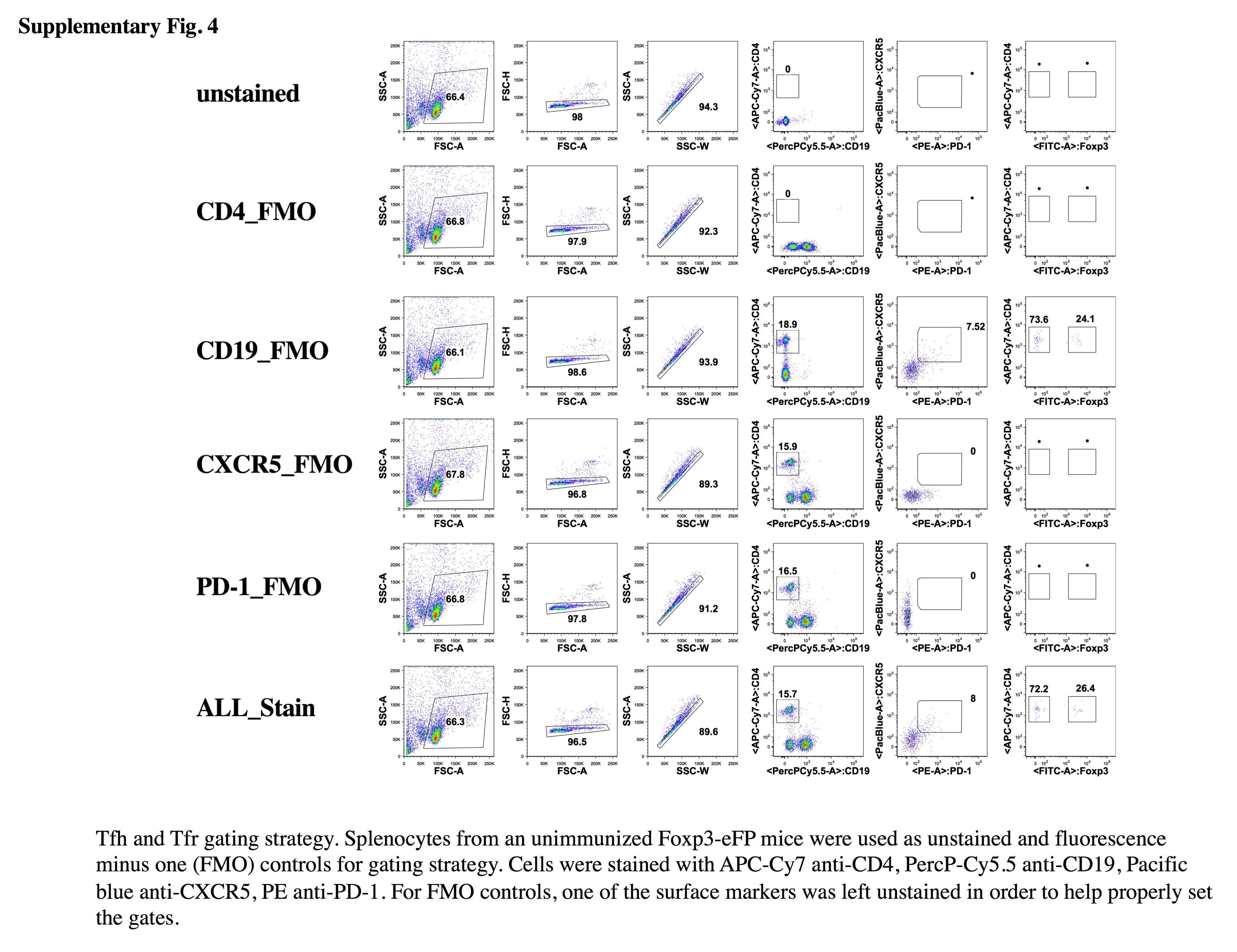

Supplement: Supplementary file 4 [file Image_4.TIFF]
